# Increasing retention of HIV positive pregnant and breastfeeding mothers on option-b plus by upgrading and providing full time HIV services at a lower health facility in rural Uganda

**DOI:** 10.1186/s12889-019-7280-5

**Published:** 2019-07-15

**Authors:** Enosk Mirembe Masereka, Tom Denis Ngabirano, Charles Peter Osingada, Christine Sekaggya Wiltshire, Barbara Castelnuovo, Agnes N. Kiragga

**Affiliations:** 1 Health Department, Ntoroko District Local Government, P. O. Box 568, Fort Portal, Uganda; 2 Department of Nursing, College of Health Sciences, Makerere University, P.O. Box 7062, Kampala, Uganda; 3 Department of Nursing and Midwifery, Mountains of the Moon University, P.O. Box 837, Fort Portal, Uganda; 4 Infectious Diseases Institute, College of Health Sciences, Makerere University, P. O. Box 22418, Kampala, Uganda

**Keywords:** Retention, Option-B plus, PMTCT, Rural, Uganda

## Abstract

**Background:**

Despite advancement in Prevention of Mother to Child Transmission (PMTCT) services, the rate of MTCT of HIV in sub-Saharan Africa is still high. This is partly due to low retention of HIV positive mothers in HIV care. We sought to determine the level of retention and the factors associated with retention among HIV positive pregnant and breastfeeding mothers following accreditation of an antiretroviral therapy (ART) clinic to offer full time ART services in one of the lower health facilities in rural Western Uganda.

**Methods:**

This study was a mixed methods study conducted in 5 health centres in rural Western Uganda from 10th April to 10th May 2017. A total of 132 retained and non-retained HIV positive pregnant and breastfeeding mothers were recruited. A Mother was categorized as retained if she had not missed her ART appointments at antenatal or postnatal clinic for ≥3 consecutive months. Questionnaires were administered and four focus group discussions were held. We used descriptive statistics to understand characteristics of mothers and their levels of retention. Thematic analysis was used to analyze qualitative data.

**Results:**

About a third (35.6%) of the mothers were aged 18–24 with a median age of 26 (IQR 23, minimum age of 16 and maximum age of 39). More than half, 73 (55.3%) of all mothers were in HIV care for 3–24 months and about 116(87.9%) of all mothers were retained in HIV care. This was an improvement from 53% reported in 2015. We found lack of formal education, lack of disclosure of HIV status to the spouse, perceived lack of confidentiality and self stigmatization as factors hindering retention. The desire to have an HIV free baby, fear of death and opportunistic infections, support from significant others and community groups were factors associated with retention.

**Conclusions:**

We observed improved retention in lower health centres and to achieve 100% retention, we recommend interventions such as sensitizing HIV positive mothers on disclosure of HIV status to spouse, maintaining confidentiality of client information at the clinic, support to girl child education and formation of community support groups.

**Trial registration:**

This study was retrospectively registered with the Uganda National Council for Science and Technology (UNCST), registration receipt number 10961 on the 9th March, 2018.

## Background

Globally, an estimated 1,400,000 HIV infected women give birth with almost 330, 000 babies becoming infected with HIV annually [1]. About 91% of these reside in sub-Saharan Africa [1]. The transition from giving a single dose of zidovudine to mothers during pregnancy and labor to a currently more effective lifelong triple combination of antiretroviral drugs (option-B plus) has registered tremendous success in Prevention of Mother to Child Transmission (PMTCT) rates [2].

In sub-Saharan Africa, with improved PMTCT strategies, the number of newly infected infants decreased by 58% [3]; however, despite the wide scale-up, coverage and benefits associated with PMTCT services, retention of mothers in care is still a challenge. For example in Malawi, although the option B-plus program showed an overall increase in coverage of antiretroviral therapy (ART) for PMTCT, only 42% of mothers were retained in care in high volume facilities in the first 3 months period following initiation of ART [4]. Drug side effects, partner support, the desire to prevent transmission and improve health were cited as influencers of mothers’ retention in HIV care [5].

In Uganda, about 5.5% of pregnant mothers are HIV positive, of which 85% access ART as part of Antenatal Care (ANC) services. Although 97% of pregnant mothers attend the first ANC visit, only 48% complete the recommended 4 visits and the trend of ANC attendance mirrors the trend of retention of HIV positive mothers on option-B plus [6]. The roll out of option-B plus in Uganda in 2012 resulted in an increase in the number of mothers initiated on ART, however retention of mothers in HIV care progressively declines [6]; with only 79, 70 and 56% returning for ART drug refill at 1 month, 3 months and 6 months [7]. Health service providers’ attitudes, stigma, discrimination, low ART stock levels and lack of means of transportation of clients to nearby health facility are some of the factors that hinder retention in HIV care [8].

Despite efforts to increase retention in HIV care through integrating PMTCT in postnatal care, follow-up phone calls and home visits; mothers continue to disengage from HIV care in rural western Uganda. In the study area, retention of HIV positive pregnant and breastfeeding mothers has been as low as 53% [9] and ART services including PMTCT have been previously offered at only four ART accredited health facilities leaving majority of the HIV positive mothers un-served.

This study aimed at determining the level of retention and the factors influencing retention among HIV positive pregnant and breastfeeding mothers following accreditation of an ART clinic to offer comprehensive full time ART services at one of the lower level health centres in rural Western Uganda.

## Methods

### Study design

This study was a mixed methods study that employed a descriptive cross sectional study design and a phenomenological study design for collection of both quantitative and qualitative data.

### Study area and population

This study was carried out in antenatal and postnatal care clinics at 5 ART accredited health facilities in rural western Uganda. The study included HIV positive pregnant and breastfeeding mothers receiving HIV care as part of antenatal care (ANC) and postnatal care (PNC) at 5 (five) study sites. The study was conducted from 10th April to 10th May, 2017, 6 months after the accreditation of a lower level health facility to offer comprehensive full time HIV/AIDS treatment, care and support services. HIV positive pregnant and breastfeeding mothers who were enrolled into HIV care for at least 3 months were eligible for this study.

### Sample size determination and participant selection

The sample size for the quantitative aspect of this study was determined using the Leslie Kish survey sampling formula [10]; *Z* (the value from standard normal distribution) at 95% Confidence Interval (CI) was set at 1.96. *P,* the proportion of HIV positive mothers not retained in HIV care in rural Uganda was 47% [9]; *e* (the margin of error) was set at 5% (0.05) to arrive at *n* (the actual sample size) of 383 respondents. Given *N* (the sampling frame) of 200 HIV positive pregnant and breastfeeding mothers for this study, *n* was adjusted to obtain a probability proportionate to size sample, *n*_*o*_. Using a formula for small finite populations and considering the finite population correction factor (considering *n =* 383 and *N* = 200), *n*_*o*_ of 132 HIV positive pregnant and breastfeeding mothers was considered.

At every study site, we compiled lists of HIV positive mothers in care. Their addresses and appointment dates were extracted from health records stored in the onsite HIV clinical data base. The expected mothers to attend at the clinic per day at every study site were listed. Mothers were then consecutively recruited into the study as they came to the clinic for their subsequent visits on their appointment dates at their respective health facilities; those who missed turning up on their appointment dates and did not still turn up within the next 2 days were tracked through their peer mothers and Village Health Teams (VHTs) and interviewed from their homes. This was done until a total sample size of 132 HIV positive mothers was realized. Four Focus Group Discussions (FGDs) with 8 purposively selected retained and non- retained HIV positive mothers per FGD were held at 4 study sites. A total of 32 participants were involved in the qualitative aspect of the study. Those who participated in the quantitative aspect of the study were not involved in the qualitative study. The remaining 36 of 200 participants were those who were not eligible for the study and those who were lost to follow up.

### Data collection

Quantitative and qualitative data collection was performed sequentially. Quantitative data was collected using a questionnaire formulated purposely for this study. Questions on retention in HIV care such as number of visits attended in the past 3 months and others on socio-demographic characteristics of mothers were asked. A mother was categorized as retained if she had not missed her ART appointments at antenatal or postnatal clinic for ≥3 consecutive months. Qualitative data collection was guided by FGD guide that consisted of open ended questions. During the FGDs, we broadly asked questions on individual, site level and community facilitators and barriers to retention in HIV care. We posed a question and allowed the group members to give their opinions on the matter in details without interruption; however prompts to obtain more information were introduced appropriately in the discussion. We used probes such as silence, urging phrases and nonverbal attending skills. We conducted FGDs until we realized saturation point of information. FGDs were recorded using tape recorder. We also wrote down the participants’ narratives in note books.

### Data analysis methods

Quantitative data was analyzed using SPSSv23. Mothers’ demographic characteristics and levels of retention were analyzed using descriptive statistics. Data from focus group discussions were organized into manageable narratives; this was followed by a search for key concepts and patterns in the narratives. The generated patterns were transformed into themes. The themes and narratives were used to triangulate the quantitative findings.

### Ethical considerations and protection of study participants

Approval from Institutional Review Board (IRB) at the School of Health Sciences (SHS), Makerere University was obtained. The study was registered with the Uganda National Council for Science and Technology (UNCST). Written consent was obtained from all study participants.

## Results

### Socio-demographic characteristics

The median age of the respondents was 26 years (Inter-quartile range 23, minimum age of 16 and maximum of 39), 105(79.5%) were breastfeeding and 27(20.5%) were pregnant. Of those recruited into the study, 96(72.8%) were living within 5 km radius from the nearby health facility. A significant proportion, 41(31.1%) of mothers reported being inconvenienced by geographical obstacles such as floods, lakes and hills that exist between their residence and the health centre. More than half, 73(55.3%) of the mothers were in HIV care for 3–24 months while 59(44.7%) were in HIV care for more than 24 months [Table 1].

**Table 1:** Socio-demographic characteristics of HIV positive pregnant and breastfeeding mothers in rural Uganda

Socio-demographic characteristics	Frequency (*n*_*o*_ = 132)	Percentage
Category of respondents		
Breastfeeding mothers	105	79.5
Pregnant mothers	27	20.5
Pregnancy trimesters		
First	2	1.5
Second	16	12.1
Third	9	6.8
Marital status		
Married	90	68.2
Not Married	42	31.8
Tribe		
Mutuuku	53	40.2
Mutooro	41	31.1
Mukonzo	21	15.9
Others	17	12.8
Education level		
Never went to school	39	29.5
Primary	85	64.4
Secondary	8	6.1
Religion		
Christian	111	84.1
Muslim	21	15.9
Distance to health centre		
0-5Km	96	72.8
> 5Km	36	27.2
Presence of geographical obstacles		
Yes	41	31.1
No	91	68.9
Nature of obstacles		
Floods	16	12.1
Hills	8	6.1
Game park	6	4.5
Swamps and lake	11	8.3
Years in HIV care		
0.3–2	73	55.3
> 2	59	44.7

### Level of retention of HIV positive pregnant and breastfeeding mothers in HIV care in rural Uganda

**Fig. 1:**
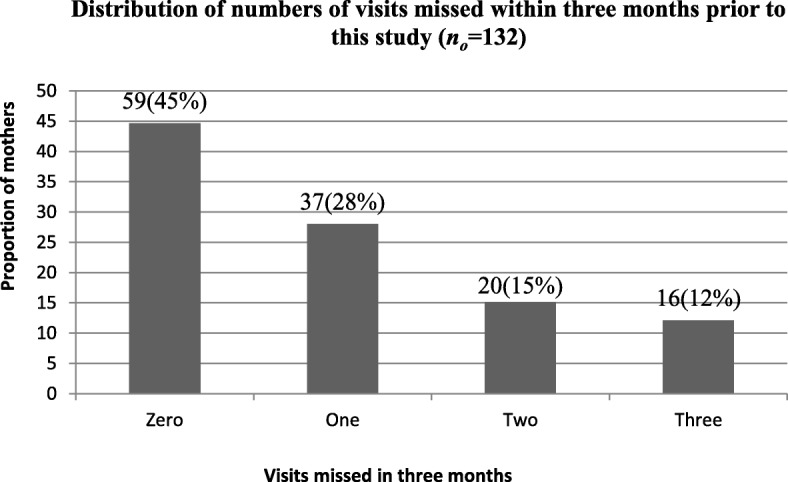
Distribution of numbers of visits missed by HIV positive pregnant and breastfeeding mothers within 3 months prior to this study

In this study, about 116(87.9%) of the HIV positive mothers were retained in HIV care, however 73 (55.3%) missed at least one visit in the last 3 months that preceded this study (Fig. 1).

### Qualitative findings of factors influencing retention of HIV positive pregnant and breastfeeding mothers in rural Uganda

Factors associated with retention were the desire to have an HIV free baby, fear of death and opportunistic infections, support from significant others and community groups.

*“I fear to be asked by my young son wanting to know how and from where he got the illness….”*

*“….. One can even die so fast if the virus is too much because it is growing every day.…….”*

*“I refill my drugs because I fear TB (Tuberculosis) and Kisipi (herpes zoster)”.*

*“…..In my village, there is a post test group called X, they help by reminding us to pick and take drugs. …….”*

Individual and site level factors that hinder retention in HIV care were self-stigmatization, inability to read, non-disclosure of HIV status by HIV positive mothers to their spouses and lack of organized systems at clinics.

*“…..when the nurse writes the next appointment date in my book and I am not told verbally, because I cannot read, when I inquire after some time from my colleague, she tells me, you know what? Your appointment date already passed………..”*

*“…..I used to get ashamed and asking within myself that how will people see me, the daughter of so and so picking drugs…….”*

*“The time when I used to miss coming to pick my drugs was when I got another man (husband). ……. my new husband did not know my HIV status ….”*

*“…..there is one time when I came to the health centre during antenatal, I was number one to arrive and so other mothers found me and the line became long. As more mothers came in, my book kept on being placed the last and others being added on it. I stayed for a very long time…”*

The community level factors that hinder mothers’ retention in HIV care included rumor mongering, fear of being rejected by the husband and coming from the same village as health workers working at the health centre.

*“……at my village, there are women who can rumor monger, they count your movements, even going to town; they say she has gone to pick drugs. … when one looks good, they say they are the drugs she is taking that make her look good……”*

*“……. For me personally I fear some staffs because we come from the same village. The other health workers are okay……..”*

*“…….I have my friend who never wanted her husband to know her status, when he knew, they separated. Currently she has another man (husband). Because of the fear to separate with the new husband, she has stopped the drugs”*

## Discussions

In this study, about 87.9% of HIV positive mothers were retained in HIV care. This level of retention was higher than the 53% that was reported in 2015–2016 [11]. It was cited that the desire to keep healthy and have an HIV free baby, fear of death, fear of opportunistic infections and support from significant others were reasons for the high retention of mothers in HIV care. Although most were retained, more than half of the HIV positive mothers missed at least one visit in 3 months that preceded this study. This trend suggests that retention in care may decrease over time as demonstrated by national data where the number of HIV positive mothers who returned for refill of ART drugs showed a declining trend from 79 to 56% in 1–6 months after initiation of ART [7].

During FGDs, mothers who had no formal education and could not read and write were found to be more likely to miss their appointments and less likely to be retained. Delvaux et al. reported that women with no formal education are unlikely to take note of their appointment dates, understand most of the teaching concerning their health, know the importance of maintaining consistence with appointments and unlikely to make rightful choices [12]. In a qualitative interview, getting into a new sexual relationship was found to hinder retention in HIV care more especially when the mother had not disclosed her HIV status to the new partner. Lack of disclosure usually leads to taking of drugs in hiding and where hiding becomes difficult, appointments and drugs are missed all together [13]. Similarly shame of being seen picking drugs, a form of self stigmatization was a barrier to retention in HIV care. McMahon and colleagues reported anticipated stigma among HIV positive women who have not disclosed their HIV status and fearing that others would learn of their HIV status as one of the common reasons for developing shame and being less likely to be retained in HIV care [13]. Rumor mongering at community level was frequently cited as a barrier to retention in HIV care. Mothers cited some of their community members as being unhelpful as they kept talking about them. McMahon and colleagues reported this as one reason for mothers to limit their movements including limiting going to the clinic for ART and other services [13]. On the other hand, this could be a form of perceived lack of confidentiality where mothers think that information that pertain their HIV status has leaked to the community and people are talking about them. The fear of confidentiality breach among clients is a commonly pronounced barrier to retention in HIV care. Therefore keeping clients’ information confidential greatly improves their retention in HIV care [14].

## Conclusions

In this study, we observed a relatively high level of retention in HIV care among rural HIV positive pregnant and breastfeeding mothers. Similar to previous studies, we observed that retention is influenced by individual, site and community level factors. To achieve 100% retention of HIV positive mothers in HIV care in rural areas of Uganda, we recommend comprehensive interventions including sensitizing HIV positive mothers on being consistent with their visits to the clinic, disclosure of HIV status to the spouse, maintaining confidentiality at the clinic, forming community support groups and support to girl child education.

### Strengths of this study

This was a mixed methods study; this allowed us obtain extra information from participants. The qualitative data was used to triangulate the findings obtained from the quantitative study. The questions about quality of service delivery in health facilities were asked. Both Retained and non- retained clients were recruited into the study.

### Study limitations

The study relied on responses from pregnant and breastfeeding mothers and some of these might have been affected by recall bias. The interviewers endeavored to clearly articulate the questions to ensure that the mothers responded accurately. However, with the low levels of education observed, this might have affected some of the responses. Due to a small sample size involved in this study, quantitative analysis was basically descriptive and we could not go further to run multivariate logistic regression models.

## Data Availability

All data and materials for this study shall be availed whenever requested by editorial team, reviewers and other users. The data set can be accessed by sending a request to mirembeenos@gmail.com

## Abbreviations

ANC: Antenatal Care
ART: Antiretroviral Therapy
CHS: College of Health Sciences
FGD: Focus Group Discussion
IRB: Institutional Review Board
PMTCT: Prevention of Mother to Child Transmission
UNCST: Uganda National Council for Science and Technology
VHT: Volunteer Health Team
